# A New Sesquiterpene from the Fruits of *Daucus carota* L.

**DOI:** 10.3390/molecules14082862

**Published:** 2009-08-03

**Authors:** Hong-Wei Fu, Lin Zhang, Tao Yi, Jing-Kui Tian

**Affiliations:** Zhejiang Provincial Key Laboratory for Chinese Medicine Screening, Exploitation & Medicinal Effectiveness Appraisc for Cardio-cerebral, Vascular & Nervous System, The Key Laboratory of Biomedical Engineering Ministry of Education, China; Department of Biomedical Engineering, Zhejiang University, Hangzhou 310027, China; E-mail: zxxtjk@gmail.com (H.W.F.)

**Keywords:** *Daucus carot*, umbelliferae, sesquiterpene

## Abstract

Phytochemical investigation of the fruits of *Daucus carota* L. resulted in the isolation of a new sesquiterpene named as daucucarotol (**1**). Its structure was elucidated on the basis of 1D and 2D NMR experiments, coupled with MS studies. To our knowledge, compound **1** is the first example for a natural eudesmane sesquiterpene with a hydroxymethyl group located at a methine carbon rather than a usual quaternary carbon in the two fused six-membered ring systems.

## Introduction

*Daucus carota* L. (Umbelliferae) is a biennial herb, which is widely distributed throughout the World. The fruits of the plant (common name: wild carrot fruits) have been used in Traditional Chinese Medicine for the treatment of ancylostomiasis, dropsy, chronic kidney disease and bladder afflictions [1], due to a wide range of reported pharmacological effects, including antibacterial [2], antifungal [3], anthelmintic, hepatoprotective [4] and cytotoxic [5] activities. The presence of sesquiterpenes [6,7,8], chromones [9], flavonoids [10,11], coumarins [6,12] and anthocyanins [13,14] have been demonstrated in previous chemical investigations. Its potential medicinal importance and our interest in the chemistry of bioactive constituents [15,16,17,18], prompted us to initiate a chemical investigation of this plant. This paper deals with the isolation and structure elucidation of the new sesquiterpene **1** from the fruits of *D. carota* L., along with its cytotoxic activities against two human gastric cancer cell lines BGC-823 and AGS. 

## Results and Discussion

### Structure Elucidation

**Figure 1:**
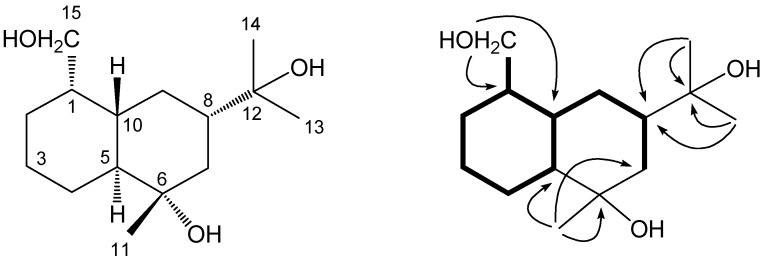
Structure, key H-^1^H COSY (─) and HMBC correlations (H→C) of compound **1**.

Purification of the 95% EtOH extract from the fruits of *D. carota* L., using combinations of silica gel, ODS and Sephadex LH-20 column chromatography, gave one new sesquiterpene **1** (Figure 1), which structure was completely established by various spectroscopic analyses and MS studies.

**Figure 2:**
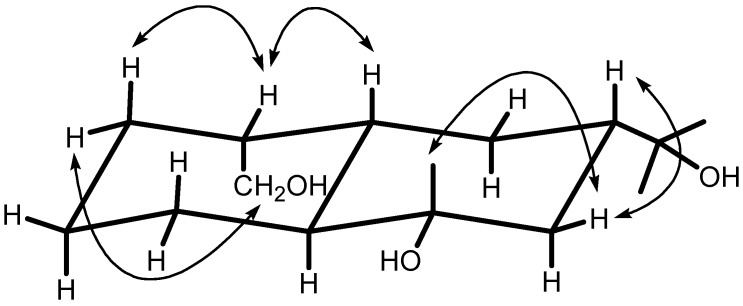
Key NOESY correlations (H→H) of compound **1**.

Compound **1** (44.0 mg, yield: 0.022 %) was obtained as a colorless oil, [α]_D_^22^ +14.6° (MeOH; *c* 0.7). Its molecular formula was determined as C_15_H_28_O_3_ by HRESIMS (*m/z* 279.1930 [*M*+Na]^+^). The ^13^C-NMR data, in combination with analyses of the DEPT and HMQC spectra, revealed the presence of three tertiary methyls, six methylenes (one of which was oxygenated), four methines and two quaternary carbons (which were all oxygenated). Further, the ^1^H-NMR spectrum showed a set of signals including three tertiary methyl signals at *δ*_H_ 1.14 (3H, s, Me-11), 1.13 (3H, s, Me-13) and 1.10 (3H, s, Me-14), and one hydroxymethyl signals at *δ*_H_ 3.68 (1H, dd, *J* = 10.6, 6.2 Hz, H-15) and 3.42 (1H, dd, *J* = 10.6, 8.4 Hz, H-15). The three oxygenated carbon signals in the ^13^C-NMR spectrum (at *δ*_C_ 73.2, 75.2 and 63.9 ppm) were assigned to C-6, C-12 and C-15, respectively. The large C7–C8–C9–C10–C1(­–C15)–C2–C3–C4–C5 partial structure unit was deduced from the detailed analyses of ^1^H-^1^H COSY and HMQC spectral data of **1** (Figure 1). Interpretation of the HMBC spectral data led to the connectivities of the partial unit mentioned above and tertiary methyls coupled with quaternary carbons to construct the planar structure of compound **1**. The HMBC correlations from *δ*_H_ 1.13 (Me-13) to *δ*_C_ 50.4 (C-8) and 75.2 (C-12), as well as from *δ*_H_ 1.10 (Me-14) to *δ*_C_ 50.4 (C-8) and 75.2 (C-12), indicated the existence and location of the isopropyl group at C-8. The attachment of the remaining methyl at C-6 was deduced on the basis of the HMBC correlations from *δ*_H_ 1.14 (Me-11) to *δ*_C_ 55.6 (C-5), 73.2 (C-6) and 46.6 (C-7). The stereochemistry of **1** was confirmed by careful assignment of NOESY data (Figure 2). The NOESY correlation between *δ*_H_ 1.70 (H*_α_*_ax_–5) and *δ*_H_ 2.36 (H*_β_*_ ax_–10) was not observed, indicating that the A/B-ring linkage was a trans-configuration. The *α*-orientation of a hydroxymethyl group was suggested by the NOESY correlations for CH_2_*_α_*_ ax_-15 (*δ*_H_ 3.68, 3.42)/H*_α_*_ eq_–2 (*δ*_H_ 1.36), H*_β_*_ eq_–1 (*δ*_H_ 2.15)/H*_β_*_ ax_–2 (*δ*_H_ 1.70) and H*_β_*_ ax_–10 (*δ*_H_ 2.36). It was also supported by no NOESY correlation between CH_2_*_α_*_ ax_-15 and H*_β_*_ ax_–10. Furthermore, the NOESY correlation between H*_β_*_ ax_–7 (*δ*_H_ 1.85) and H*_β_*_ eq_–8 (*δ*_H_ 1.56) indicated the isopropyl group was *α*-orientated. In addition, the *β*-orientation of Me-11 and the *α*-configuration of OH–6 were determined by the NOESY correlation between Me*_β_*_ ax_-11 and H*_β_*_ eq_–7. Based on the above results, the structure of **1** was established as (1α,5α,8α,10β)-decahydro-6α-hydroxy-8α,8α,6β-trimethyl-1,8-naphthalene-dimethanol, which is a new compound that we have named daucucarotol. 

**Table 1:** ^1^H- and ^13^C-NMR Data (500 and 125 MHz, resp.; CD_3_OD) of **1**. *δ* in ppm, *J* in Hz.

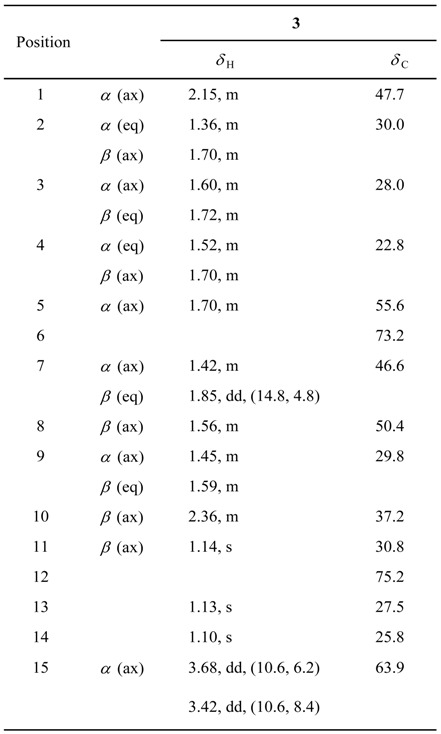

### Cytotoxicity Assay

Cytotoxicity is commonly used as a target for screening anticancer compounds and the MTT assay is commonly employed. The new eudesmane type sesquiterpene **1** was screened for cytotoxicities against two human gastric cancer cell lines BGC-823 and AGS at concentration of 100 μg/mL. The results indicated that compound **1** displayed weak cytotoxic activity against BGC-823 with inhibition of 13.3%, but showed less cytotoxicity against AGS. 

## Experimental

### General

Optical rotations were measured using a JASCO DIP-370 digital polarimeter in a 0.5 dm length cell. ^1^H- and ^13^C-NMR were recorded using a Bruker ARX-500 and ARX-125 MHz NMR spectrometer with TMS as the internal reference, and chemical shifts are expressed in *δ* (ppm). HRESIMS was taken on a LCQ DECA XP plus spectrometer. D_101_ (Chemical Plant of Nankai University, Tientsin, China), silica gel (200-300 mesh, Qingdao Haiyang Chemical Co. Ltd., China), Sephadex LH-20 (Amersham Pharmacia Biotech) and ODS (35-50 μm, Alltech) were used for column chromatography (CC). TLC was performed with silica gel GF254 pre-coated plates (Qingdao Haiyang).

### Plant Material

The fruits of *D. carota* were purchased in September 2007 in Hangzhou, Zhejiang Province, P. R. of China, and identified by one of the authors (L.Z.). A voucher specimen was deposited in the Herbarium of the College of Biomedical Engineering and Instrument Sciences, Zhejiang University, China.

### Extraction and Isolation

The air-dried fruits of *D. carota* L. (2.0 kg) were refluxed twice with 95% aqueous EtOH. The combined EtOH extracts were concentrated, and then partitioned between CHCl_3_ and H_2_O. The CHCl_3_ layer (38.4 g) was subjected to silica gel CC with a gradient of petroleum ether-EtOAc to give three fractions (1–3). Fraction 3 (5.7 g) was purified by silica gel CC [petroleum ether/EtOAc (1:1)] and Sephadex LH-20 CC with MeOH to give **1** (44.0 mg).

### Cytotoxicity Assay

The two human gastric cancer cell lines BGC-823 and AGS were maintained in RPMI 1640 medium supplemented with 10% fetal bovine serum, 100 U/mL penicillin and 100 μg/mL streptomycin at 37 °C under a humidified atmosphere of 95% air and 5% CO_2_. The cell growth inhibition was assessed *in vitro* by a colorimetric MTT assay, as previously reported [19].

## Conclusions

A new eudesmane type sesquiterpene **1** was isolated from the fruits of *D. carota* L. To our knowledge, compound **1** is the first example for a naturally occurring eudesmane sesquiterpene with a hydroxymethyl group on a methine carbon and not a usual quaternary carbon in the two fused six-membered ring systems. This finding may provide a hint to identify similar structures in the future.

## Supplementary Files

Supplementary File 1
