# Fu *et al.* A New Sesquiterpene from the Fruits of *Daucus carota* L. *Molecules* 2009, *14*, 2862-2867

**DOI:** 10.3390/molecules14103868

**Published:** 2009-09-28

**Authors:** Hong-Wei Fu, Lin Zhang, Tao Yi, Jing-Kui Tian

**Affiliations:** 1 Department of Biomedical Engineering, Zhejiang University, Hangzhou 310027, China; E-mail: zxxtjk@gmail.com (H-W.F.); 2 Zhejiang Provincial Key Laboratory of Chinese Medicine Screening, Exploitation and Medicinal Effectiveness Appraisal for Cardio-Cerebral Vascular and Nervous System, The Key Laboratory of Biomedical Engineering of the Ministry of Education, Hangzhou 310027, China; E-mail: zhanglin@zju.edu.cn (L.Z.)

We realized that the affiliations of the authors were incorrectly listed in our paper published in Molecules recently [1]. The correct affiliations are the following:

**Hong-Wei Fu ^1,2^, Lin Zhang ^1,2^, Tao Yi ^1^ and Jing-Kui Tian ^1,2^**

^1^  Department of Biomedical Engineering, Zhejiang University, Hangzhou 310027, China; E-mail: zxxtjk@gmail.com (H-W.F.)
^2^  Zhejiang Provincial Key Laboratory of Chinese Medicine Screening, Exploitation and Medicinal Effectiveness Appraisal for Cardio-Cerebral Vascular and Nervous System, The Key Laboratory of Biomedical Engineering of the Ministry of Education, Hangzhou 310027, China; E-mail: zhanglin@zju.edu.cn (L.Z.)
