# Supplementary material for: A New Sesquiterpene from the Fruits of Daucus carota L
Source: Molecules. 2009 Aug 3;14(8):2862–7. doi: 10.3390/molecules14082862 (PMC6255428; doi:10.3390/molecules14082862)
Supplement: Supplementary File 1 [file molecules-14-02862-s001.pdf]

Molecules **2009**, *14*, 3868; doi:10.3390/molecules14103868

OPEN ACCESS

molecules

ISSN 1420-3049

www.mdpi.com/journal/molecules

Correction

Fu *et al.* A New Sesquiterpene from the Fruits of *Daucus carota* L. *Molecules* 2009, *14*, 2862-2867

Hong-Wei Fu [1,2], Lin Zhang [1,2], Tao Yi [1] and Jing-Kui Tian [1,2,]*

[1] Department of Biomedical Engineering, Zhejiang University, Hangzhou 310027, China; E-mail: zxxtjk@gmail.com (H-W.F.)

[2] Zhejiang Provincial Key Laboratory of Chinese Medicine Screening, Exploitation and Medicinal Effectiveness Appraisal for Cardio-Cerebral Vascular and Nervous System, The Key Laboratory of Biomedical Engineering of the Ministry of Education, Hangzhou 310027, China; E-mail: zhanglin@zju.edu.cn (L.Z.)

* Author to whom correspondence should be addressed; E-mail: tjk@zju.edu.cn; Tel.: +86-571-88208454; Fax: +86-571-87951091

Received: 25 September 2009 / Published: 28 September 2009

We realized that the affiliations of the authors were incorrectly listed in our paper published in Molecules recently [1]. The correct affiliations are the following:

Hong-Wei Fu [1,2], Lin Zhang [1,2], Tao Yi [1] and Jing-Kui Tian [1,2]

[1] Department of Biomedical Engineering, Zhejiang University, Hangzhou 310027, China; E-mail: zxxtjk@gmail.com (H-W.F.)

[2] Zhejiang Provincial Key Laboratory of Chinese Medicine Screening, Exploitation and Medicinal Effectiveness Appraisal for Cardio-Cerebral Vascular and Nervous System, The Key Laboratory of Biomedical Engineering of the Ministry of Education, Hangzhou 310027, China; E-mail: zhanglin@zju.edu.cn (L.Z.)

References and Notes

1. Fu, H.-W.; Zhang, L.; Yi, T.; Tian, J.-K. A New Sesquiterpene from the Fruits of *Daucus carota* L.. *Molecules* **2009**, *14*, 2862-2867.

© 2009 by the authors; licensee Molecular Diversity Preservation International, Basel, Switzerland. This article is an open-access article distributed under the terms and conditions of the Creative Commons Attribution license (http://creativecommons.org/licenses/by/3.0/).
